# Supplementary figures and images for: Harnessing Wolbachia cytoplasmic incompatibility alleles for confined gene drive: A modeling study
Source: PLoS Genet. 2023 Jan 23;19(1):e1010591. doi: 10.1371/journal.pgen.1010591 (PMC9894560; doi:10.1371/journal.pgen.1010591)

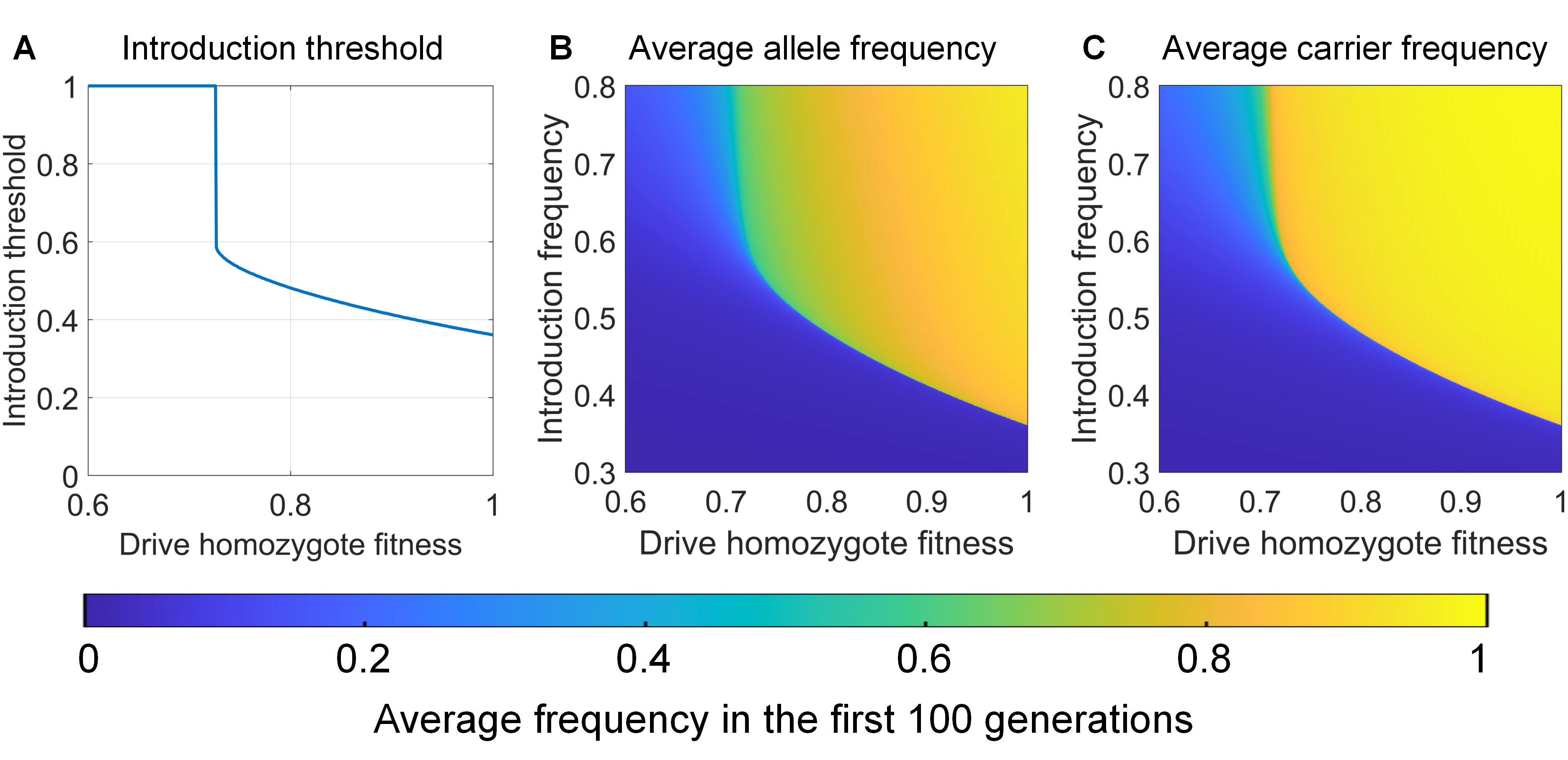

Supplement: S1 Fig — A: The introduction threshold as a function of drive homozygote fitness. The drive allele is lost for any introduction frequency below 1 when fitness is less than 0.72. B,C: Average drive allele and carrier frequencies in the first 100 generations after releasing drives for each set of parameters. (TIF) [file pgen.1010591.s001.tif]

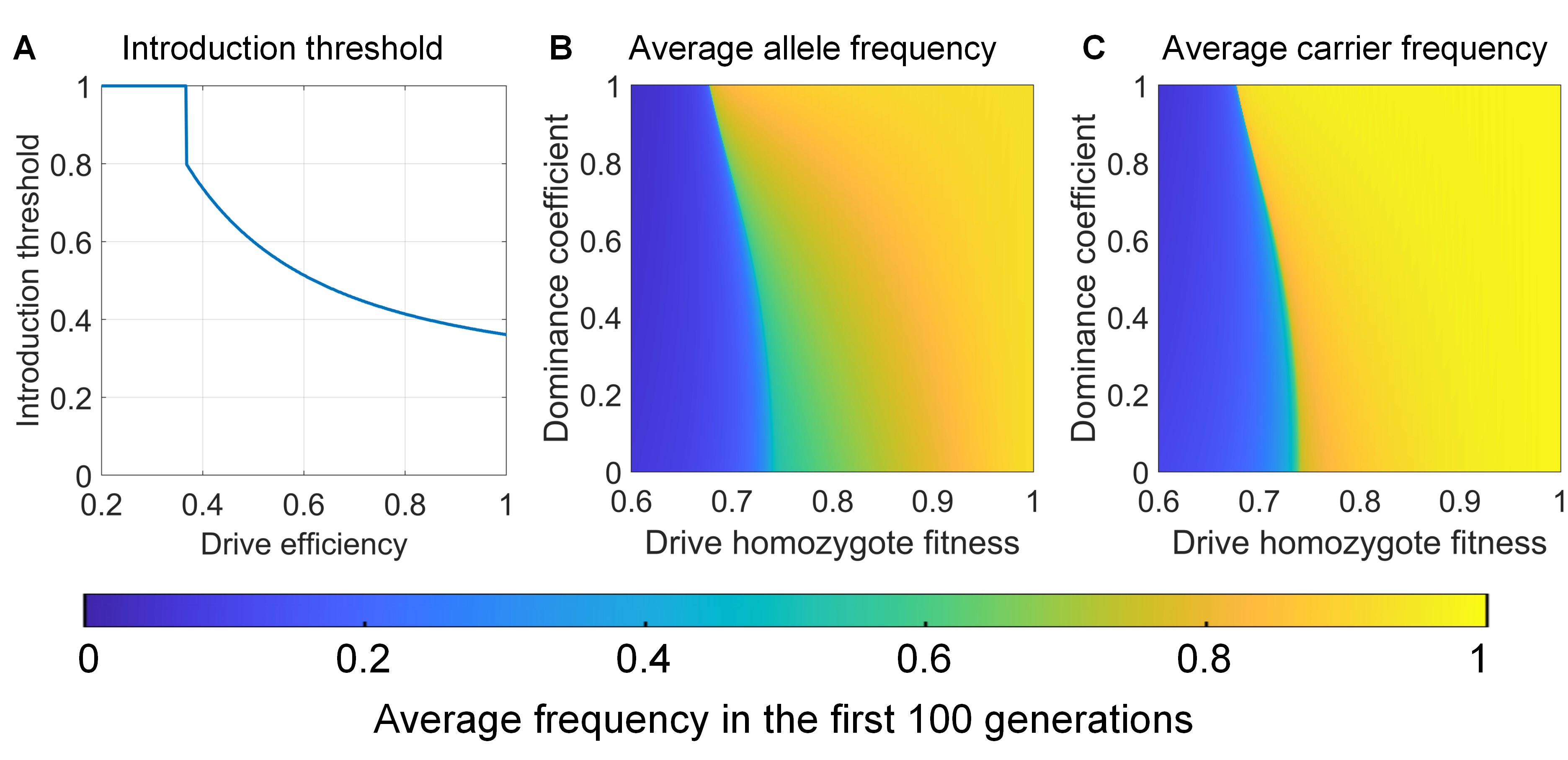

Supplement: S2 Fig — A: The introduction threshold as a function of drive efficiency, which is the value of both toxin and antidote efficiency. The CifAB allele is lost for any introduction frequency below 1. B,C: Average drive allele and carrier frequencies in the first 100 generations after releasing drives for each set of parameters. (TIF) [file pgen.1010591.s002.tif]

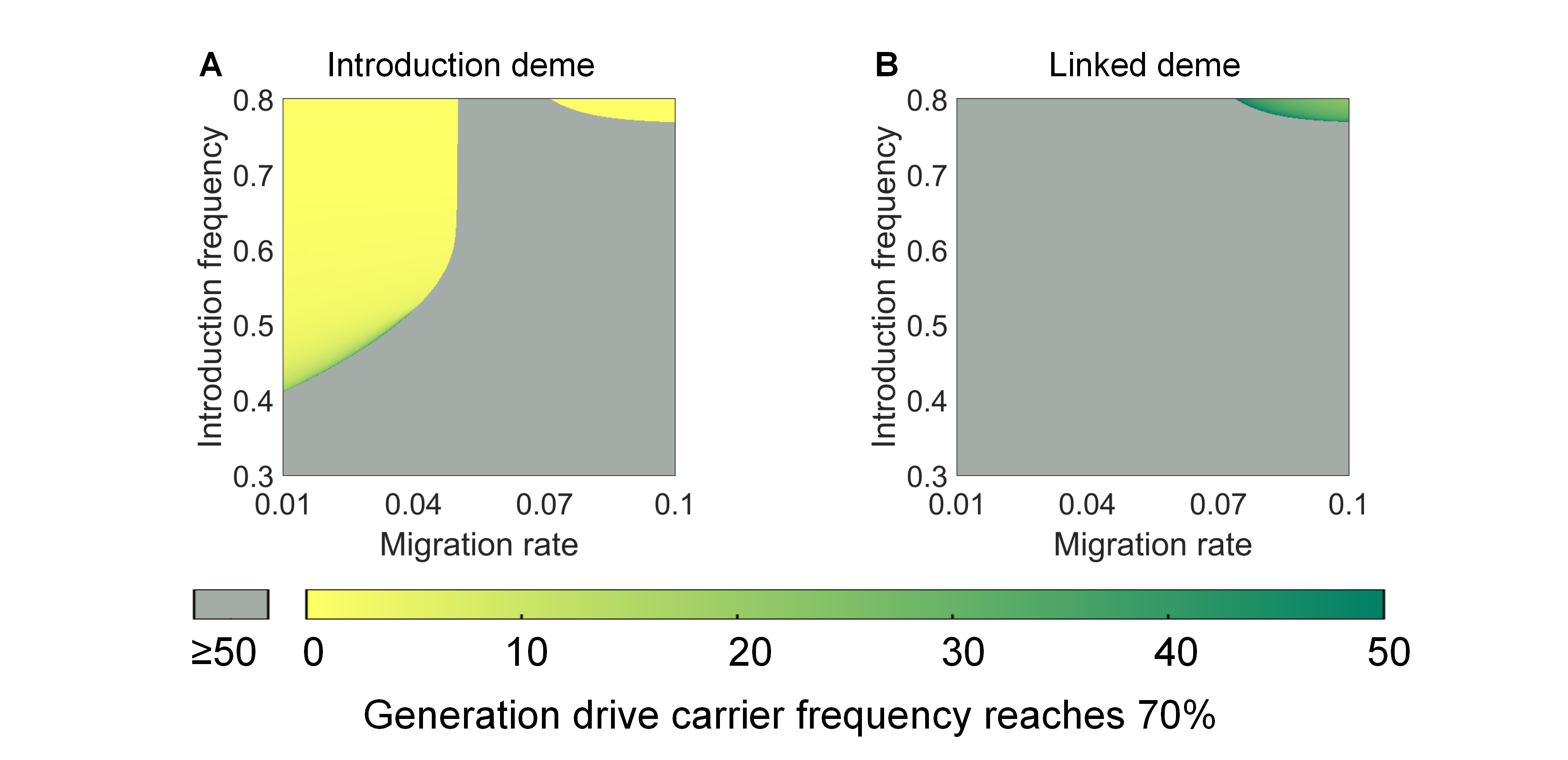

Supplement: S3 Fig — In 2-deme scenarios, the first generation when drive carrier frequency reaches 70% is collected in A: introduction deme and B: linked deme. Gray indicates that drive is lost or carrier frequency is unable to reach 70% in the first 50 generations after drive release. (TIF) [file pgen.1010591.s003.tif]

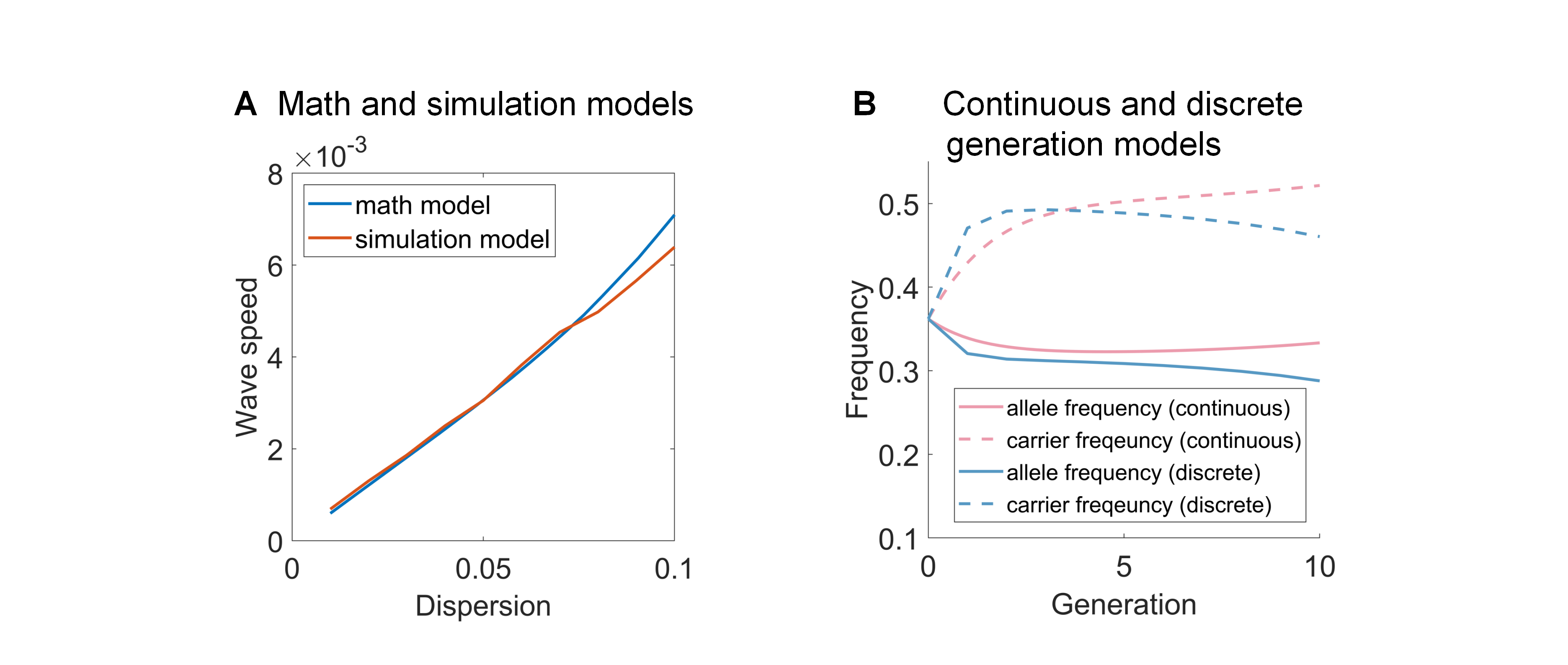

Supplement: S4 Fig — A: Wave speed measurements in spatial math and simulation models essentially match when dispersion factor v is below 0.07. The wave speed in the models has a slight difference when dispersion is increased due to stochastic effects (a larger region of low drive frequency at higher dispersion increases this effect). B: Frequency trajectories of the continuous and discrete-generation mathematical models with the same release conditions, showing an example where results substantially differ between the models near critical release values. This is because in the first few generations, allele frequency may decrease more in the discrete-generation model, making the drive allele more likely to get lost in the population. (TIF) [file pgen.1010591.s004.tif]

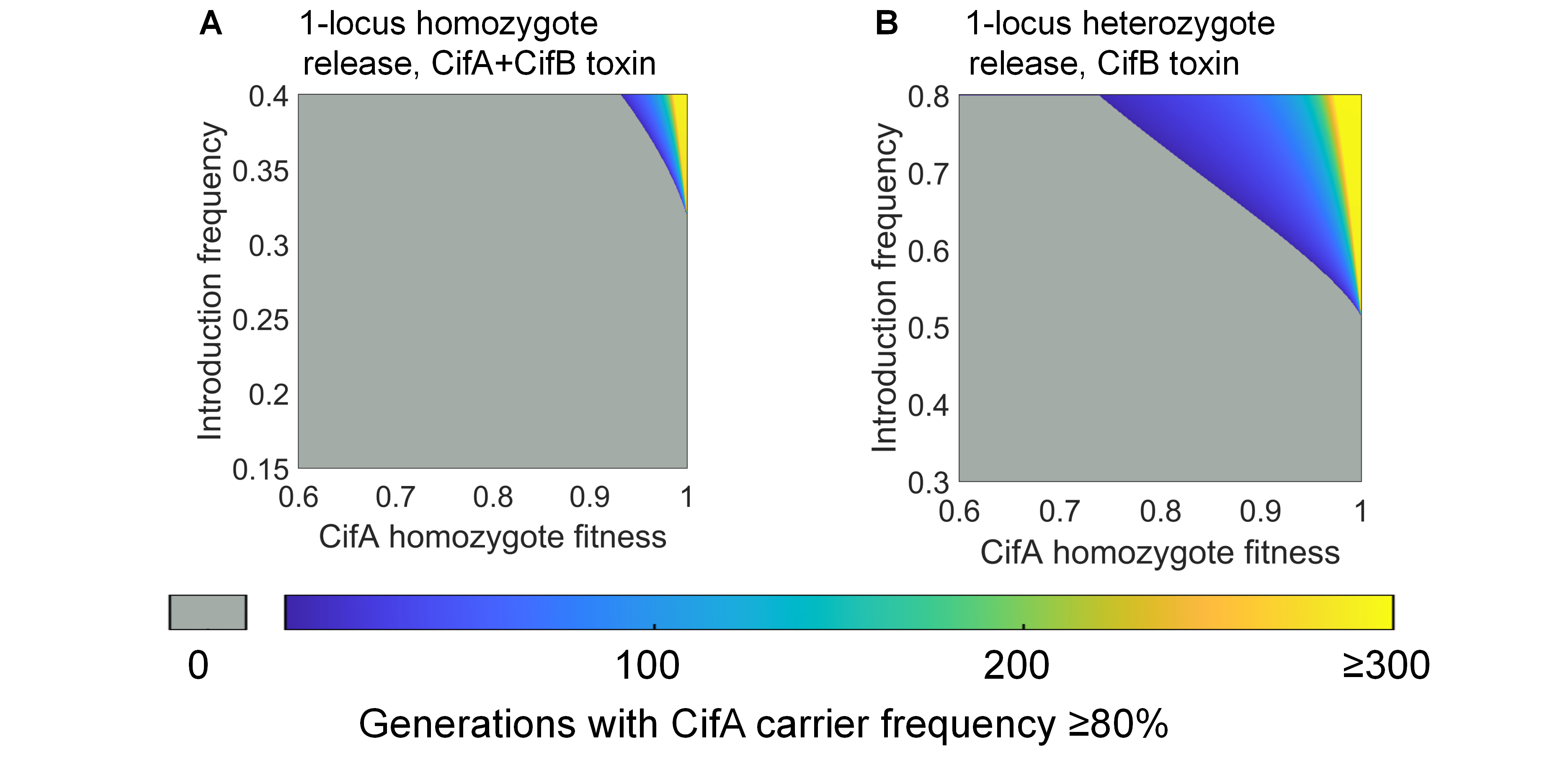

Supplement: S5 Fig — We model scenarios where cifA and cifB alleles must share the same locus. A: A 1-locus homozygote release with both cifA and cifB is required for the toxin. Both cifA/cifA homozygotes and cifB/cifB homozygotes are introduced at the specified introduction frequency. B: A 1-locus cifA/cifB heterozygote release, and only cifB is needed for the toxin effect. Gray indicates that the drive carrier frequency is unable to reach 80%. (TIF) [file pgen.1010591.s005.tif]

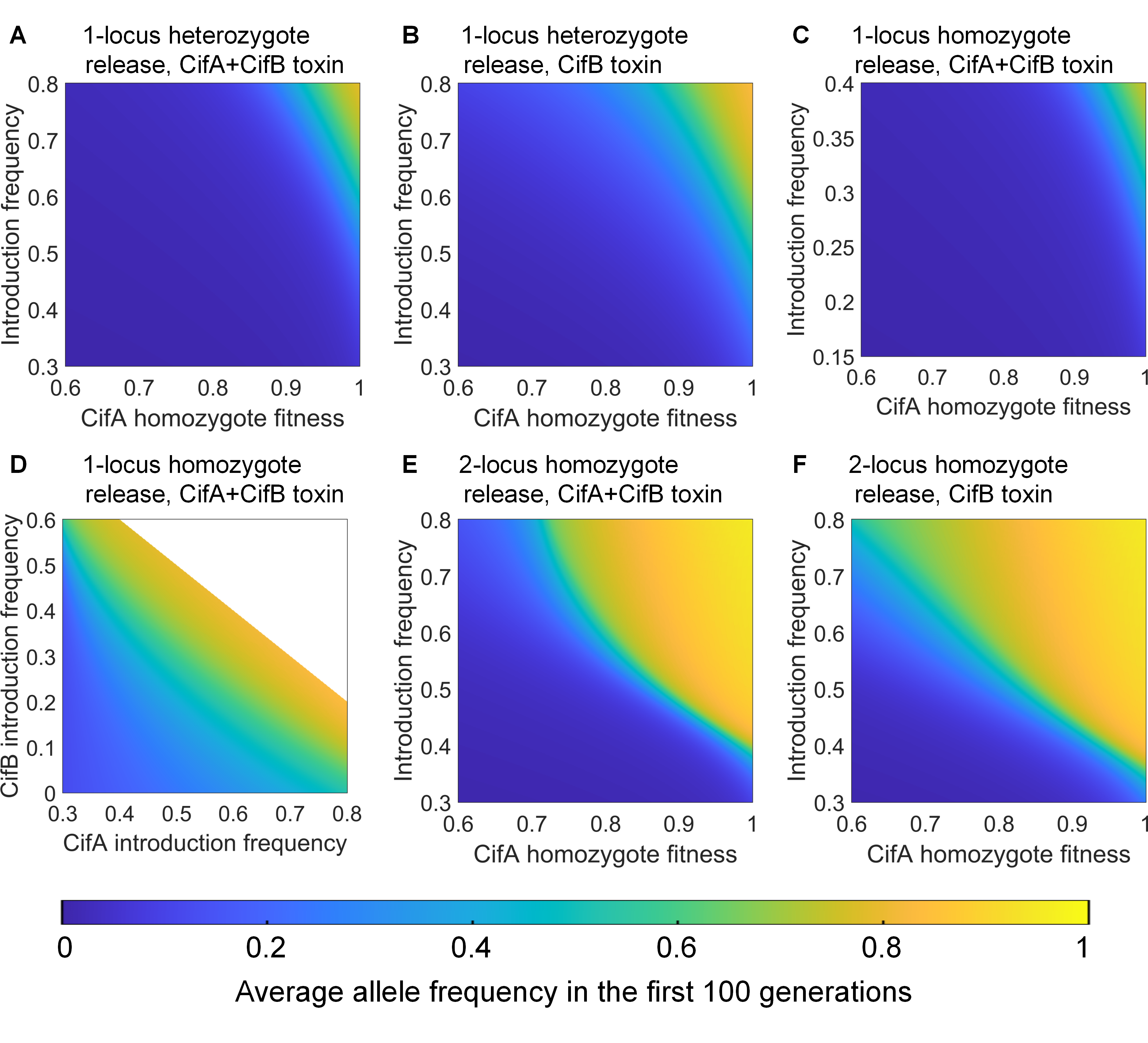

Supplement: S6 Fig — All homozygous fitness costs are on the cifA allele and are 0.95 unless otherwise specified. A: A 1-locus cifA/cifB heterozygote release. B: A 1-locus cifA/cifB heterozygote release, and only cifB is needed for the toxin effect. C: A 1-locus homozygote release. Both cifA/cifA homozygotes and cifB/cifB homozygotes are introduced at introduction frequency. D: A 1-locus homozygote release. E: A 2-locus homozygote release. F: A 2-locus homozygote release, and only cifB is needed for the toxin effect. White indicates regions of parameter space that are impossible (the total fraction of starting drive individuals cannot exceed 1). (TIF) [file pgen.1010591.s006.tif]

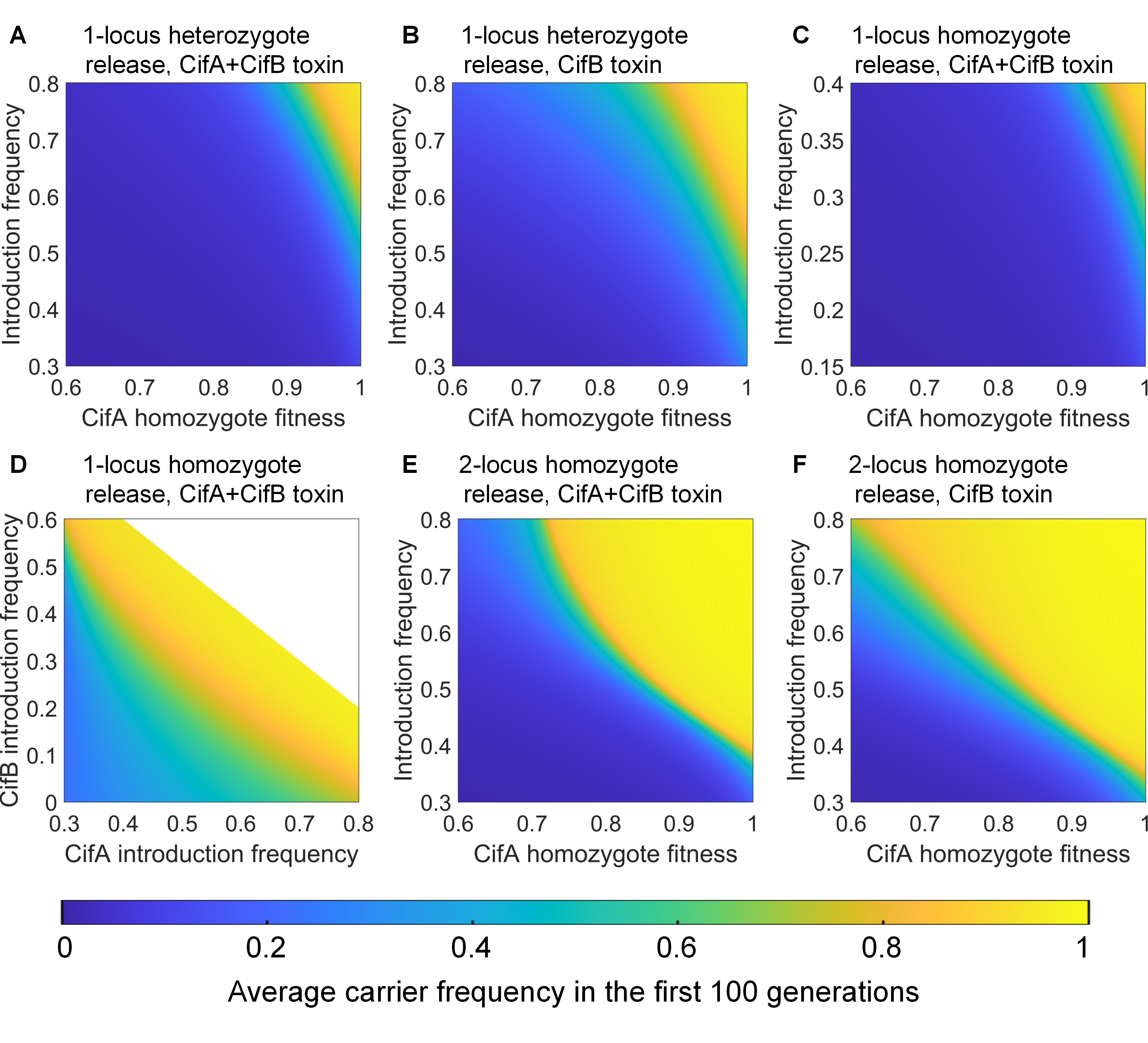

Supplement: S7 Fig — All homozygous fitness costs are on the cifA allele and are 0.95 unless otherwise specified. A: A 1-locus cifA/cifB heterozygote release. B: A 1-locus cifA/cifB heterozygote release, and only cifB is needed for the toxin effect. C: A 1-locus homozygote release. Both cifA/cifA homozygotes and cifB/cifB homozygotes are introduced at introduction frequency. D: A 1-locus homozygote release. E: A 2-locus homozygote release. F: A 2-locus homozygote release, and only cifB is needed for the toxin effect. White indicates regions of parameter space that are impossible (the total fraction of starting drive individuals cannot exceed 1). (TIF) [file pgen.1010591.s007.tif]

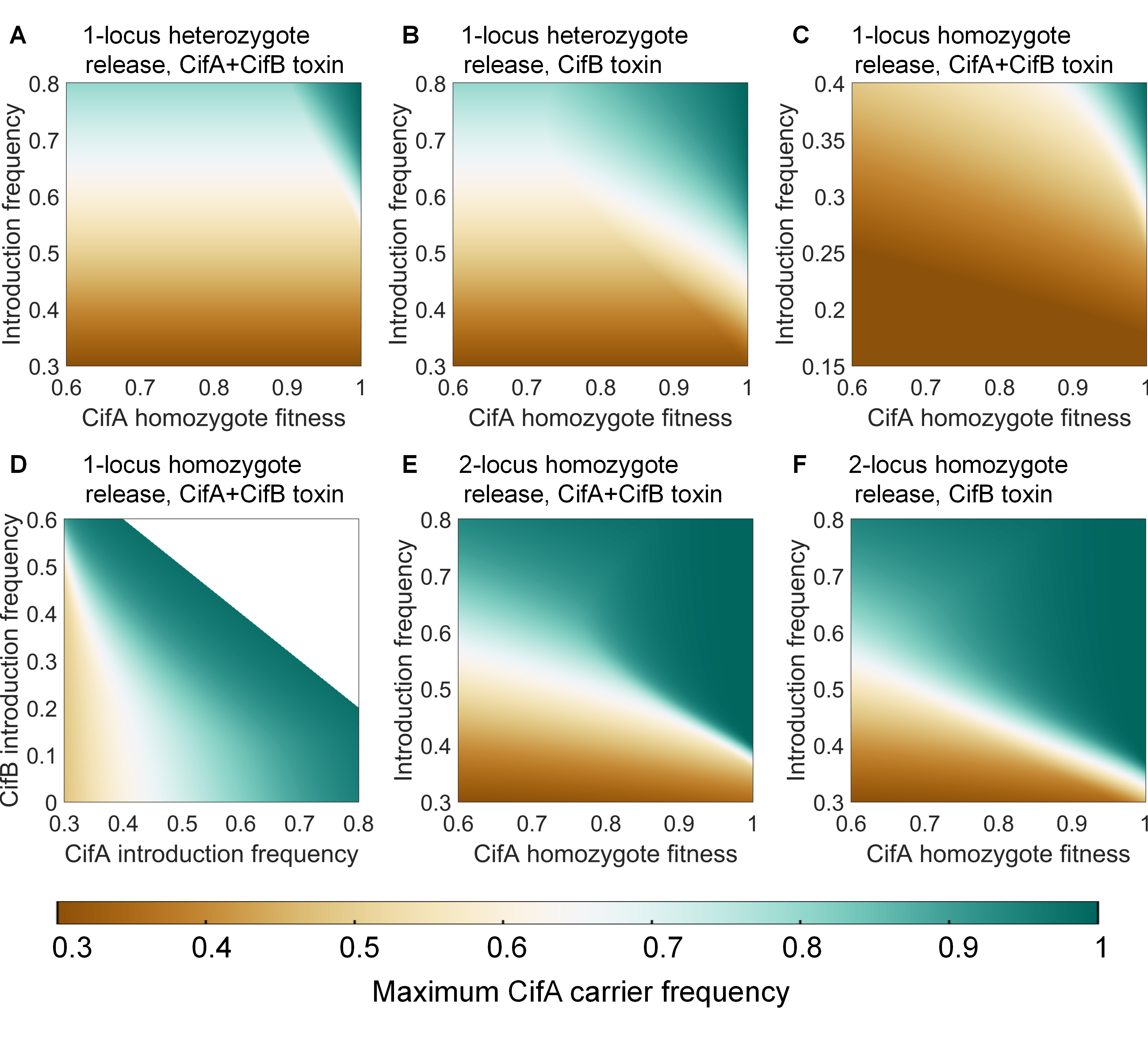

Supplement: S8 Fig — All homozygous fitness costs are on the cifA allele and are 0.95 unless otherwise specified. A: A 1-locus cifA/cifB heterozygote release. B: A 1-locus cifA/cifB heterozygote release, and only cifB is needed for the toxin effect. C: A 1-locus homozygote release. Both cifA/cifA homozygotes and cifB/cifB homozygotes are introduced at introduction frequency. D: A 1-locus homozygote release. E: A 2-locus homozygote release. F: A 2-locus homozygote release, and only cifB is needed for the toxin effect. White indicates regions of parameter space that are impossible (the total fraction of starting drive individuals cannot exceed 1). (TIF) [file pgen.1010591.s008.tif]

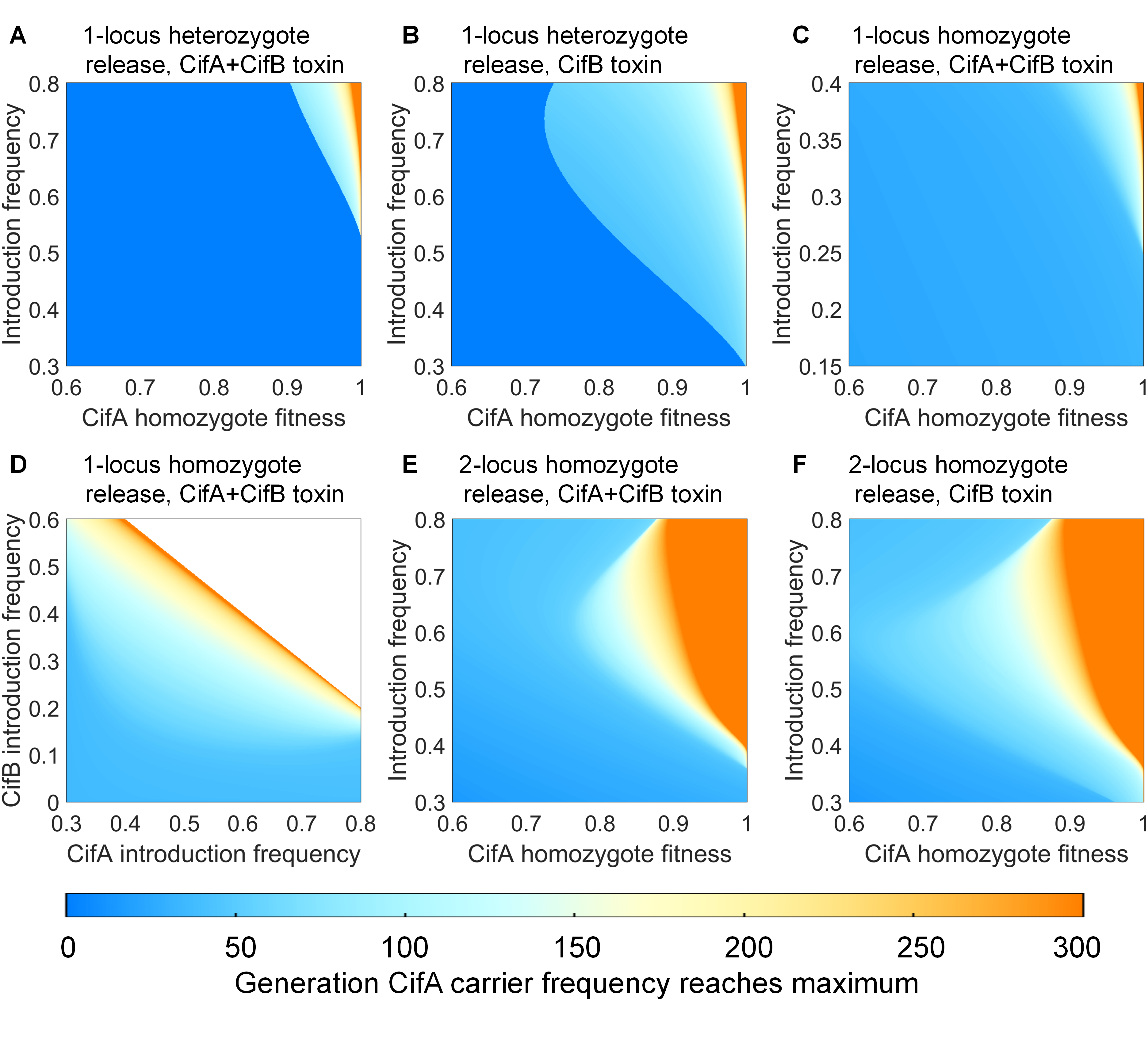

Supplement: S9 Fig — All homozygous fitness costs are on the cifA allele and are 0.95 unless otherwise specified. A: A 1-locus cifA/cifB heterozygote release. B: A 1-locus cifA/cifB heterozygote release, and only cifB is needed for the toxin effect. C: A 1-locus homozygote release. Both cifA/cifA homozygotes and cifB/cifB homozygotes are introduced at introduction frequency. D: A 1-locus homozygote release. E: A 2-locus homozygote release. F: A 2-locus homozygote release, and only cifB is needed for the toxin effect. White indicates regions of parameter space that are impossible (the total fraction of starting drive individuals cannot exceed 1). (TIF) [file pgen.1010591.s009.tif]
