# Supplementary material for: Harnessing Wolbachia cytoplasmic incompatibility alleles for confined gene drive: A modeling study
Source: PLoS Genet. 2023 Jan 23;19(1):e1010591. doi: 10.1371/journal.pgen.1010591 (PMC9894560; doi:10.1371/journal.pgen.1010591)
Supplement: Attachment — Submitted filename: Response to Reviewers.pdf [file pgen.1010591.s010.pdf]

Dear Editor, thank you for handling our manuscript. We have addressed all the comments of the reviewers below and hope that the manuscript is now ready for publication.

Sincerely,
Jiahe Li and Jackson Champer

Reviewer #1: "Harnessing Wolbachia cytoplasmic incompatibility alleles for confined gene drive: a modeling study" builds off three important advances from recent years: the expansive literature on synthetic drive systems, Wolbachia's deployment as a tool in vector control worldwide (reliant on CI and pathogen interference traits), and the discovery of CI's genetic basis. CI (or cytoplasmic incompatibility) is a conditional sterility where embryos die when infected males fertilize uninfected eggs but not infected eggs.

This study mathematically assess how Wolbachia's CI genes (cifA and cifB) could be used as a gene drive system encoded in the host genome. In this scenario, the cifAB drive would be paired with a payload that confers resistance to a disease of interest and then spreads that resistance into a vector population. I don't have a complete grasp of the mathematics underlying their analyses and cannot effectively comment on their accuracy. However, I am confident that the broader principles of their analyses are well reasoned, and their analyses are thorough, with some gaps. Below, I highlight three larger issues and provide some specific line-by-line feedback.

1) Literature review – The literature review for this paper could be vastly improved. This is particularly relevant to the Wolbachia literature from which the foundations of this paper are manifest. In the line-by-line feedback I provide some specific recommendations for papers to cite. However, I would encourage the authors to broadly review the literature on CI as a mechanism for spread, Wolbachia as a tool for vector control, and CI's mechanistic underpinnings. For instance, surprisingly missing, but highly relevant, is a 1999 study from Turelli and Hoffmann in Insect Molecular Biology. They also model how CI transgenes in the host nuclear genome could be used for drive but of course do this without knowing the specific details of the CI genes.

For Turelli and Hoffman 1999, we were not aware of this study before (coming from a gene drive rather than Wolbachia background), but we now prominently reference it at the end of the introduction, as well as later in the results where we compare our introduction frequency to their haploid model.

We have also followed nearly all of the reviewer's below recommendations for adding more literature (we thank the reviewer for thoroughly helping us with these references). However, we are hesitant to go much beyond this. Our introduction already needs to combine literature from gene drive, confinement, *Wolbachia*, and cytoplasmic compatibility, making it already longer than usual. A completely comprehensive review on *Wolbachia* and cytoplasmic incompatibility may thus be a little beyond the scope of this manuscript (though we believe that with the recent explosion in cytoplasmic incompatibility mechanism literature, a review article focusing relating this and other *Wolbachia* work would be quite timely).

2) Comparisons to cytoplasmic drive – This manuscript provides a rather thorough investigation of the parameter space for a transgenic cifAB gene drive. However, no effort is made to formally compare this drive mechanism to the natural cytoplasmic drive. Given that the cytoplasmic drive is currently being deployed and protects millions from disease, it makes sense that cifAB gene drive should not only be compared to other gene drive systems but also to the "wild-type" system it would aim to replace or complement. This could be addressed by adding a description of the ways that the models for spread differ when the cif genes are encoded in the host vs the Wolbachia genome and adding analyses where the cifs are Wolbachia encoded.

We agree that there is more room for comparison between CifAB drive and *Wolbachia* population dynamics. However, *Wolbachia* have been extensively modeled, and we feel that new modeling for *Wolbachia* is therefore out of the scope of this paper. CifAB drive might use the same mechanism, but because it is on the genome, its populations dynamics are just as different from *Wolbachia* is from other engineered gene drives.

We now offer some additional insight near the end of the first results paragraph, which now reads,
"In contrast, male drive carriers have a lower chance of successful reproduction compared to male wild-type individuals, especially if the drive carrier frequency is low, thus reducing the frequency of the drive in the next generation due to removal of paternal drive alleles. Therefore, there exists an introduction threshold for this type of drive, below which the drive will be eliminated. This is not the case for *Wolbachia* bacteria, which lack an introduction threshold in ideal form and only gain a threshold if there are fitness costs or imperfect transmission efficiency. This is because *Wolbachia* bacteria are maternally transmitted, so no *Wolbachia* transmission is lost when infected males fail to produce offspring with wild-type females."

We have also expanded our discussion (4th paragraph in the discussion section, which already makes some comparisons) comparing CifAB drive to *Wolbachia* in a similar manner and added the following additional material:
"Depending on the species, it may be easier to modify its genome than to infect it with *Wolbachia*, or at least *Wolbachia* that are incompatible with those that can already be found in a species. CifAB could also be more easily used to modify a population that is infected with a

different (and perhaps unwanted) *Wolbachia* strain because a new *Wolbachia* strain in this instance would have a 50% introduction threshold if equal to the existing strain."

3) Fitness advantage – Numerous analyses consider the impacts of the relative fitness of the drive strain relative to the wild type. However, these studies assume that the drive strain has an equal or lower fitness than the wild type. While this assumption is reasonable in many scenarios, it remains plausible that the drive strain could have a fitness advantage that would considerably impact the spread dynamics and have implications for its role as a confined gene drive. There are cases in the text where this is described verbally, but the analyses could be improved by displaying a fitness advantage scenario.

In general, a cargo with a fitness advantage could spread on its own, without a gene drive (though a drive could still be used for confinement in such situations). It is also unclear if any cargos under consideration for most drive applications would provide more than a transient advantage, and the literature of modeling gene drives thus generally assumes that the drive can't confer a fitness benefit to its host. Modeling such a benefit would create any fundamentally new patterns unless the fitness advantage was very large.

Line-by-line feedback:
Abstract sentence 1 – 'Wolbachia' is plural.
Abstract sentence 3 – 'alternative mechanism' would be more accurate as 'in other systems'. It's still debated whether it's truly an alternative mechanism.
Line 120 – I recommend removing reference 42 and adding Hoffmann 1990, Kriesner 2013, and Kriesner 2016 that describe the parameters that govern Wolbachia's spread.
Line 141 – The work from Verily should also be cited here. For example, Crawford et al 2020 and Beebe et al 2021. Both focus on IIT in Aedes aegypti.
Line 162 – The first letter of the Wolbachia strain name should be italics. For instance, in wMel, the w is italics.
Line 163 – Utarini et al. 2021 should be cited. They show that Wolbachia releases in Yogyakarta inhibit local dengue spread by over 85%.
Line 165 – It's more accurate to say "a pair of genes commonly associated with phage WO." There are rare exceptions where they are not in the phage.
Line 168 – Specifically, Drosophila melanogaster. Also, the way this is written needs to be revised slightly since it implies that whether one or both genes are required is host dependent, but it's more complex than that. For instance, Sun et al. 2022 showed CifB from wNo Wolbachia is sufficient to cause transgenic CI in D. melanogaster. In reality it seems that CifA's requirement in CI might be dependent on both Wolbachia and host genotypes. For simplicity it's possible to say something like "transgenic expression of cifB is sometimes sufficient to cause CI, though co-expression of cifA and cifB is required in other systems".

Line 172 – Reference 53 isn't particularly relevant to this statement.

All of these have been addressed according to the reviewer's recommendation.

Line 176 – Needs a few sentences or paragraph describing the rescue side of things. Later it is mentioned that CifA rescues CI, but background isn't provided. Shropshire et al. 2018 and 2019 show CifA rescues CI and Xiao et al. 2021 and Wang et al. 2022 suggest that CifA rescues CI by binding CifB.

We now provide a little more background on this and appropriate references.

Line 191 – A figure, or part of a figure, illustrating the different strategies could be helpful.

We now reference Figure 1A here, which has also been expanded.

Line 193 – When referencing a gene, the first letter of the symbol should be lower case and the symbol italicized (e.g., cifA and cifB, in italics). As written (CifA and CifB) it implies a protein is being discussed. This is an error throughout the paper.

Gene name italics have been corrected.

Line 209 – There is a robust literature from Turelli, Barton, and others on the parameters that govern Wolbachia's spread. This paper seems an excellent opportunity to lay out the different assumptions underlying Wolbachia's spread via CI and CifAB gene drive.

We have added some additional references a couple subsections later when we discuss Wolbachia. As noted by the reviewer, this literature is quite robust with many variations and species-specific models covering much more detail than our own single study. We thus reference these, but don't necessarily explicitly describe all of them, especially because many parameters considered are quite specific to the *Wolbachia* bacteria themselves, rather than general mechanisms of their cytoplasmic incompatibility.

Line 289 – Cite literature on efficiency variation. Shropshire et al. 2020 eLife reviews the literature on this topic. Also, Ritchie et al 2022 looked at the impact of temperature and male age on transgenic CI.
Fig 1A – The drive mechanism also requires that females with the drive allele can rescue, correct? This should be illustrated here for completion.

Fig 1B – While the legend says that gray means the drive allele is eliminated, the key indicates that it will spread given > 20 generations. Is the legend for Fig 1B accurate given that it tops at 20, but Fig 1D indicates that some introduction frequencies/fitness consequences take far longer than that?
Fig 1C,D – Include the fitness value in the legend or figure. In fact, when it's relevant, it would be helpful to describe the important fixed parameters in the figure or legend throughout the manuscript.

All of these have been addressed according to the reviewer's recommendation.

Line 739 – How many generations will it take for 36.051% to reach fixation? The figure shows it very slightly increasing by generation 40.

It would take about 77 generations for the carrier frequency to reach 99% (the rate of increase is slow close to the threshold, but it rapidly increases further away). However, we'd prefer not the extend the scale because the other more important frequency trajectories are scaled nicely, and this mostly-flat line makes it clear that the critical carrier frequency is 50% in Figure 1D.

Fig 2 legend – "dive" should be "drive"

Fixed.

Fig 3 – For consistency with other figures, and to make it easier to read, can gray again be used to indicate parameter space where the drive allele fails to spread? The difficult part with using blue here is the statement in the legend that says "Blue colors tend to represent". It'd be helpful to know where the line is between tend to not, and do.

We have removed the word "tend". In this graph, we are showing an average frequency over 100 generations to give an ideal of short- and mid-term performance (though long-term behavior can be understood too based on outlines). Drive failure is indicated by low but nonzero values, so it's not quite as clean a distinction as the other figures, which use a 70% threshold to clearly distinguish between success and failure. Note that we have several supplemental figures that follow this style, so it's not quite as much of an "oddball" figure as one might expect from only the main manuscript.

Line 851 – It would be valuable to consider a scenario where instead of varying introduction frequency, equilibrium carrier/allele frequency is varied relative to migration. In this scenario I'm curious what would happen if there was a linked deme with a late-

stage introduction deme with a stable drive frequency > 90%. What level of migration would be required from this stable population into the linked population to make the drive spread?

This is somewhat akin to a "migration threshold", with constant influx of drive individuals into a single focal population. Our previous work indicates that these generally follow a similar pattern to introduction thresholds, so a full analysis of these with all of our variables would not be expected to produce significantly different findings. However, we did calculate the migration threshold of an ideal drive and have added the following text to the end of the "2-deme" section: "A similar scenario involves a single deme with a continuous influx of drive individuals, presumably from a separate deme that does not receive any migration from the focal deme. In this case, a drive homozygote influx of approximately 4.5% of the total population per generation will be sufficient to eventually allow the drive to fully spread through the deme. At lower levels of migration, a low equilibrium will be reached with the drive allele frequency staying below 15\%."

Line 917 – It would help to have context for what a wave speed of these magnitudes would mean in a natural context. It's hard to assess this without a deeper comparison of the simulated arenas relative to a wild population.

In this case, our model is abstract, with the scale of the model set by the dispersion parameter. This would be different for each species (which can var over several orders of magnitude), as well as various ecological factors. Thus, we cannot provide a scale for this study. Future, species-specific models including this type of drive should certainly attempt to do so.

Line 1048 – It's relevant to cite literature from Turrelli (Turelli 1994, for example).

We now cite this paper in the introduction, but this line is about self-limiting variants of CifA and CifB (which has more in common with killer-rescue drives than normal *Wolbachia*).

Line 1229 – While the math supports them being highly confined, empirical data are needed to support the math. As such, a bit more caution is warranted here and throughout. This is especially true given that a positive fitness effect is likely to prevent this from being self-limiting.

We have adjusted our wording here and in a couple of other locations to be more cautious.

Lline 1270 – You first say "did not likely find the optimal shape" but then say "ring-like

drop with no individuals in the center is optimal.”

Fixed.

Line 1280 – Another key difference is the mechanism of inheritance.
Line 1331 – Relevant to cite the transgenic CI literature regarding promoters successfully used to induce transgenic CI. For example, Shropshire et al. 2018 and 2019 show how promoter variation impacts rescue and CI respectively.
Line 1339 – Need to cite the bidirectional CI literature. For instance, Wang et al. 2022 show that binding site variation between cifA and cifB contributes to bidirectional CI. Shropshire et al. 2021 also shows how cifs from different strains interact transgenically.

We have added these references.

Line 1440 – Notably, the systems used for transgenic CI have largely used germline promoters and thus should have minimal expression in somatic tissues.

Sometimes (nanos), but vasa is often used as well, which is known to have a fair amount of somatic expression, at least when used with transgenes. It is also unclear exactly how strong promoters need to be in the germline. Something weaker than nanos or vasa (which are quite strong in the germline) might be better, or it might be too weak...

Reviewer #2: Summary:
The authors propose new types of toxin-antidote non-suppression gene drive systems using Wolbachia CifA and/or CifB genes independent of the Wolbachia bacteria and without using CRISPR homing. They did a good job of describing its properties in detail, i.e. using deterministic/mathematical models with continuous time, stochastic simulations with discrete time, and spatially explicit deterministic and stochastic models.They showed that when introduced together, these two genes result in a confined or localised gene drive; while introduced separately the drive system becomes self-limiting or transient or temporary. They also showed that the manner of release significantly affects drive persistence in spatial context. This new drive system seems to be more feasible and potentially safer to test than homing CRISPR-based gene drives.

Major Comments:
I have no major comments doubting the mathematical models and stochastic modelling employed. As far as I can tell, the models and simulation methodologies are sound.

We thank the reviewer for these comments.

Minor Comments:
- In the abstract, it's potentially helpful to explicitly specify that Wolbachia bacteria as whole organisms are not involved in the gene drive system - just their cytoplasmic incompatibility genes.

We had made the suggested change.

- Lines 212-217: I understand on a high-level that defining a generation in this way makes sense, but is it actually equivalent or similar to that of discrete non-overlapping generations? This potentially warps generation interval right after drive introduction as population size decreases as the fitnesses of wild-type females drop.

This is an interested observation. As noted in the manuscript, we define a generation as an interval when population size is at carrying capacity. When not at carrying capacity, the situation may well change. However, this would also fully apply to discrete-time models (or even the situations approximated by discrete-generations models in some realistic cases). This is not necessarily unrealistic, and removing this would likely require complex adjustments that may make the results harder to understand (since they would represent actual density-varying generation times rather than fixed times). We'd thus prefer to keep our current definition of time and generations.

- Table 1: population size, N is not the "number" of individuals, rather the relative population size, i.e. number of individuals relative to the number of individuals at carrying capacity.

Fixed.

- Lines 587-595: What were the border conditions used: toroidal or absorbing or reflecting boundaries, etc?

We now note this later in the section when discussion offspring placement (we use reprising boundaries).

- I think I understand that you used overlapping generations in your math models because it was a straightforward extension into spatially explicit models; however, did you bother with using discrete non-overlapping deterministic models (which is a better comparison with your stochastic simulations)? Would you expect similar properties?

We expect that all of these models should converge in most details where population size is high, allowing stochastic effects to be avoided (hence the widespread use of all of them in the field). An important exception would be in early generations where the dynamics of approaching homozygote/heterozygote equilibrium may be different in discrete versus continuous models. We did investigate this a bit in Figure 7A, which compared the threshold with SLiM models, discrete-generation math models, and continuous math models, which show some interesting differences. The homozygote drive introduction threshold is actually different in continuous versus discrete math models. We don't expect any other differences of this magnitude were we to apply such a model in our other scenarios.

- Figure 1 C and D: It's probably advantageous to add vertical lines corresponding to when Hardy-Weinberg equilibrium (HWE) was reached after homozygous drive introduction so that the unstable equilibria do not look funny, i.e. kinks around the equilibria where we expect introduction frequencies equal to the equilibria to not change since they're the equilibria.

The drive actually does not reach a Hardy-Weinberg equilibrium (sort of a shame in one sense, because this would have made it a bit easier to model, and more importantly, opened up more possibilities for analytical assessment). Equilibrium is also not technically reached at a specific point, making it hard if we wanted to highly a specific time where it was reached. Of course, this is roughly where the two intermediate releases flatten out very close to each other, but there is no firm time that this occurs.

- Figure 1 C and D: Also, does it make more sense to use a unitless time axis instead of "generations" because of generation-time warping with continuous generations?
- Figure 6 B, E and F: Similar comment to Figure 1 C and D - express the x-axis as time instead of generations.

We believe that using "generations" may remain valid in some situations while also being more intuitive for readers. However, we now explicitly state in the methods that:
"Note that strictly speaking, the average generation time may therefore change in some species when population size is not at equilibrium due to variance in birth or death rates, hence our definition based on equilibrium population size."

- Additionally, is the effect of the drive on population size insignificant?

The drive, like any toxin-antidote system, would be expected to affect total population size. However, the exact response of population size to the drive is dependent on species-specific and

ecological factors, which are beyond the scope of this study. Further, this particularly population modification drive would not be expected to affect population sizes much more than others (this can be seen indirectly in Figure S1 of our latest preprint, which uses a similar density-dependent growth curve: https://www.biorxiv.org/content/10.1101/2022.11.01.514650v1). We have added the following text to the methods section:
"We note that as a population modification drive, only modest population fluctuations are expected, and these don't substantially influence the outcome in most scenarios (though slower death rates at high densities in drive release regions can improve drive performance). The density-dependent curve is fixed in all of our modeled scenarios."

- Figure 7 B: note that K-> infinity for the spatial discrete non-overlapping generations is not available due to the reaction-diffusion model used continuous time.

We now note this in the main text that describes the figure.

Reviewer #3: This paper introduces a potential new approach to creating a gene drives for the control of insect disease vectors. The idea is to utilise the genes that cause the intracellular bacteria Wolbachia to spread in many arthropod species, by a mechanism called cytoplasmic incomparability, without the necessity of introducing the entire bacterial genome. It is an appealing prospect and clearly worthy of investigation. The paper does this using a suite of mathematical models that differ in complexity from non-spatial to spatial and deterministic to a spatial stochastic simulation. This is an excellent approach to exploring the roles of different complexities and I found the model progression logical and well explained. Overall, I enjoyed reading the manuscript; it is clearly written, instructive, and makes a good case for further research into the proposed technology. I only have a few minor suggestions for the authors to consider.

We thank the reviewer for these comments.

1. The figures were generally clear and visually appealing. In figure 1 panels B,E and F, I wondered if you could have changed the scaling to emphasise the variation – e.g. 1B is almost entirely yellow with a tiny bit of green – could you reassign the scaling so there is more green?

We have adjusted the scale to better show a range of values.

2. There is an unexplained white region in fig. 6D – I presume this is meant to be yellow? (or indefinite persistence – though this would also be yellow?).

In this one, we were releasing two separate types of individuals, and they can't add up to more than 100%. The white area represents "impossible" releases that would represent more than 100% or "more" of the total population after release, as noted at the end of the figure legend.

3. You used numerical methods throughout to analyse the deterministic models (that you call "math models"), but I wondered if it might be possible to obtain analytical solutions. E.g. for the introduction threshold from the panmictic model – did you try? To be clear I think the analyses based on numerical results are insightful, yet analytical solutions might have given even a bit more insight into the interactions between model parameters.

When we embarked upon this study, we indeed hoped to present analytical results, like previous work with Wolbachia and driving Y systems. Unfortunately, CifAB drives are more complicated from a mathematical perspective because they have three genotypes (wild-type, heterozygotes, and homozygotes) that are not at Hardy-Weinburg equilibrium. This prevented us from obtaining any satisfactory analytical results. We do hold out hope that these could be obtained in the future, and we now explicitly state in the discussion that:
"Our models were necessarily simplified to present an initial introduction to the basic properties of these new drives, and indeed, further work could provide a more fundamental understanding if analytical solutions could be obtained."

4. A small typo on pg 14 line 994 – remove "is able".

Fixed.
